# Using Virtual Reality as a Tool in the Rehabilitation of Movement Abnormalities in Schizophrenia

**DOI:** 10.3389/fpsyg.2020.607312

**Published:** 2021-01-07

**Authors:** Anastasia Pavlidou, Sebastian Walther

**Affiliations:** Translational Research Center, University Hospital of Psychiatry, Bern, Switzerland

**Keywords:** movement abnormalities, schizophrenia, virtual reality, gestures, communication

## Abstract

Movement abnormalities are prevalent across all stages of schizophrenia contributing to poor social functioning and reduced quality of life. To date, treatments are scarce, often involving pharmacological agents, but none have been shown to improve movement abnormalities effectively. Virtual reality (VR) is a tool used to simulate virtual environments where behavioral performance can be quantified safely across different tasks while exerting control over stimulus delivery, feedback and measurement in real time. Sensory information is transmitted *via* a head mounted display allowing users to directly interact with virtual objects and bodies using gestures and body movements in the real world to perform different actions, permitting a sense of immersion in the simulated virtual environment. Although, VR has been widely used for successful motor rehabilitation in a variety of different neurological domains, none have been exploited for motor rehabilitation in schizophrenia. The objectives of this article are to review movement abnormalities specific to schizophrenia, and how VR can be utilized to restore and improve motor functioning in patients with schizophrenia. Constructing VR-mediated motor-cognitive interventions that can help in retaining and transferring the learned outcomes to real life are also discussed.

## Introduction

Schizophrenia is a severe disorder with devastating symptoms affecting approximately 2–3% of the general population. These symptoms can be positive (hallucinations and delusions) and/or negative (reduced social drive and affective flattening) in nature, and include disorganized behavior and thinking, impaired cognitive and social functioning, anxiety, and lack of in-sight and self-awareness (Mccutcheon et al., 2020). As a result, schizophrenia causes tremendous individual burden, reduced quality of life, occupational performance and life expectancy (between 10 and 20 years), as well as, substantial costs to society. Although movement abnormalities are a part of the earliest descriptions of schizophrenia (Walther and Strik, 2012), their relevance was often reduced and attributed to pharmacological side effects (Walther and Mittal, 2017). Over the last decade clinicians and researchers alike have renewed their interest in movement abnormalities in schizophrenia as biological relatives, clinical high-risk individuals and non-medicated patients’ also exhibit unusual movements (Mittal et al., 2006, 2008; Walther and Mittal, 2017; Walther et al., 2020e).

## Movement Abnormalities in Schizophrenia

Movement abnormalities in schizophrenia can occur spontaneously, may continue for several hours of the day and can also come and go. Clinicians have separated movement abnormalities in schizophrenia into six distinct categories (Walther and Strik, 2012). The first known as dyskinesia, is abnormal involuntary movements, which occurs as high frequent repetitive movements (Gervin et al., 1998). The second is classified as parkinsonism and includes akinesia, rigor, and tremor in the absence of an idiopathic Parkinson’s syndrome (Waddington, 2020). The third is akathisia, characterized as restlessness and inner tension. The fourth is neurological soft signs (NSS), which are a set of tests evaluating patients’ motor coordination, sequence of motor acts, and sensory integration, which are often performed worse compared to healthy controls (Whitty et al., 2009). The fifth is catatonia, which is a complex psychomotor syndrome that includes decreased, increased, and abnormal movements, disturbances of volition, and autonomous instability (Walther et al., 2019). Finally, the sixth is psychomotor slowing and it affects both fine (writing) and gross (walking) movements, facial expressions, and speech production (Morrens et al., 2007; Osborne et al., 2020). Patients with schizophrenia often suffer from multiple movement abnormalities during the course of their illness, and are thought to be predictors in the risk of developing psychosis (Walther and Mittal, 2017).

Movement abnormalities are prevalent across all stages of schizophrenia although the symptoms are not consistent across the different stages (Walther and Strik, 2012). During the early stages, there seems to be a link between the severities of movement abnormalities with the increase risk of developing the disorder. For example, at least one movement abnormality is present in 2/3rd of non-medicated first episode psychosis patients (Peralta and Cuesta, 2010). It increases drastically in chronically medicated patients, and affects almost all elderly patients with schizophrenia (Quinn et al., 2001; Walther and Strik, 2012). In addition, increased levels of dyskinesia are reported in children who exhibit symptoms of psychosis, while individuals with increased risk of psychosis have been reported to present both dyskinesia and psychomotor slowing (Kindler et al., 2016; Damme et al., 2020). Overall, these observations suggest a crucial role of movement abnormalities in the development of schizophrenia, and its importance in successfully screening and staging their presence during the course of the disorder.

## The Ramifications of Movement Abnormalities

Besides movement abnormalities being predictors for the risk of developing schizophrenia, they are also indicative of poor social and cognitive functioning, affecting the overall quality of life of patients (Putzhammer et al., 2005). For example, NSS, parkinsonism, catatonia, dyskinesia, and akathisia reported in patients with the first episode of psychosis were strongly associated with the emergence of negative symptoms, executive dysfunctioning, and poor memory abilities (Cuesta et al., 2014, 2018a,b; Walther et al., 2015; Fritze et al., 2020; Sambataro et al., 2020; Schroder and Toro, 2020). This was true in both medicated and non-medicated patients. In addition, the presence of movement abnormalities are strongly linked to impaired gesture performance in schizophrenia, an important aspect of social communication, and were shown to be directly related to poor social functioning even at 6-month’s follow-up (Walther et al., 2016). Errors during gesture performance are very frequent and consistent in schizophrenia patients (Walther et al., 2020d), as measured using the well-established Test of Upper Limb Apraxia (TULIA; Vanbellingen et al., 2010), developed along the principal domains and semantic traits of gesture performance. Gestural errors in schizophrenia often involve spatial and temporal configurations. Minor errors include movements that are too slow or hesitant and appear almost robotic-like, with reduced amplitudes, while major errors include omissions, body-part-as-object errors, extra movements, and errors in spatial orientation (Walther et al., 2013a,b, 2020b). In addition, schizophrenia patients tend to use fewer gestures during interactions with their psychiatrist or during casual conversations (Lavelle et al., 2013, 2015). Moreover, clinical high-risk patients for psychosis not only use fewer gestures during clinical interviews, they also tend to use mismatch gestures (Millman et al., 2014). This suggest that abnormal gesturing in schizophrenia is highly relevant for communication (Walther et al., 2020d).

Likewise, impaired gesture performance in schizophrenia is also associated with cognitive impairments, such as executive dysfunction (Walther et al., 2013b, 2015). In addition, schizophrenia patients also have deficits in perceiving and interpreting gestures (Bucci et al., 2008; White et al., 2016) and this deficit is linked to movement abnormalities (Walther et al., 2015). Hence, amelioration of movement abnormalities in schizophrenia has the potential to improve both social and cognitive functioning and expand patients’ quality of life.

## Treatment Options for Movement Abnormalities

To date, the standard treatment to alleviate movement abnormalities in schizophrenia exclusively relied on pharmacology. However, treatment effects have been heterogeneous. For example, some forms of movement abnormalities, such as catatonia and dyskinesia improved following the administration of antipsychotics and benzodiazepines (Peralta and Cuesta, 2010; Walther et al., 2019), while akathisia worsened (Peralta and Cuesta, 2010). This suggests that pharmacological treatments may not be ideal candidates and offer no long-term solution in taming movement abnormalities. Preliminary findings suggest non-invasive brain stimulation (NIBS) is a potential candidate in alleviating movement abnormalities in schizophrenia patients (Walther et al., 2020a,c). Several ongoing clinical trials are currently administered to further assess the efficacy of NIBS (Lefebvre et al., 2020); Personalized Non-invasive Neuromodulation by rTMS for Chronic and Treatment-Resistant Catatonia trial (RETONIC, NCT03116425), Overcoming Psychomotor Slowing in Psychosis trial (OCoPS-P, NCT03921450), and Brain Stimulation And Group Therapy to Improve Gesture and Social Skills in Psychosis trial (BrAGG-SoS, NCT04106427). Developing alternative interventions in tackling movement abnormalities may offer a better and long-term solution while addressing the concerns regarding usage of medication and their related side effects (Lieberman et al., 2005; Stegmayer et al., 2018). Successful movement production depends on the multisensory integration of different processes, thus better rehabilitative results are more likely to ensue when combining motor-socio-cognitive processes together with multimodal sensory feedback.

## Virtual Reality as a Tool for Motor Neurorehabilitation

In recent years, Virtual Reality (VR) has become a popular tool in neurorehabilitation, as it promotes motor learning (ML) and neuroplasticity (Kleim and Jones, 2008; Cho and Lee, 2013). VR provides clinicians and researchers alike with the unique ability to simulate real world scenarios. It engages multiple senses, allows complete immersion within the virtual environment using a head-mounted display, and provides users with the opportunity to interact with virtual objects and/or other virtual characters, allowing the manipulation of a controlled and flexible setting while providing rapid online feedback, optimizing ML (Slater, 2009; Perez-Marcos et al., 2012; Parsons et al., 2017). ML is a process that examines the acquisition of newly developed motor skills *via* practice and experience and evokes a permanent change in the ability to execute a movement (Levac and Sveistrup, 2014). Thus, VR as a multi-facet system, has the potential to enrich conventional therapies and provide its users with a more intensive and enjoyable alternative that can help in retaining and transferring ML to real life (Weiss et al., 2014; Perez-Marcos, 2018; Perez-Marcos et al., 2018).

Optimization of ML in VR is highly dependent on the manipulation of testing conditions that explicitly apply (i) external focus of attention, (ii) intensity, (iii) implicit learning, (iv) diversity, (v) task specificity, and (vi) real-time feedback (Table 1; Wulf et al., 1998; Levac and Sveistrup, 2014; Perez-Marcos et al., 2018). These conditions support the acquisition, retention and transfer phase of newly developed movement skills (Willingham, 1998), and provide encouraging results in the motor rehabilitation of patients with neurological disorders. We discuss the importance of each condition below and provide their effectiveness in VR motor rehabilitation.

**Table 1:** Movement learning principles for VR rehabilitation treatment.

Movement learning principles	Examples
External focus of attention	Navigating a virtual boat (Mirelman et al., 2009).
Intensity	Multiple sessions, repetitions of task (Perez-Marcos et al., 2017).
Implicit learning	Walking on treadmill while avoiding obstacles (Yang et al., 2008).
Variance	Increasing length and height of obstacles (Jaffe et al., 2004).
Task specificity	Shopping in virtual supermarket (Rand et al., 2009).
Real-time feedback	Auditory feedback when the chosen response is incorrect (Adery et al., 2018).

External focus of attention involves directing an individual’s attention to the effect of the performed movement in their environment such as: “lift your arm to touch a mark on the wall”, and has been effective in enhancing movement performance in gait, balance, and postural training (Wulf et al., 1998; Johnson et al., 2013; Park et al., 2015). In VR, Mirelman et al. (2009) opted to teach post stroke patients how to navigate a plane or a boat within a virtual environment by moving their feet, rather than teaching patients’ how to move their feet. The use of these external cues directed patients’ attention within the virtual environment and away from the performed movements improving their overall therapeutic outcome.

Treatment intensity is highly recommended to maximize therapeutic effects, as it can induce structural neural changes. Animal studies report that functional tasks repeated a minimum of 400 times prompt such changes (Birkenmeier et al., 2010). In a recent study, VR-mediated upper limb training in chronic stroke delivered a training intensity that was 10–15 times higher than that delivered in a standard clinical training (Perez-Marcos et al., 2017). Thus, VR provided large amounts of active training time and repetitions for each session, further highlighting VR’s efficiency in treatment outcomes.

Implicit learning is a form of learning that occurs without the person’s awareness (Reber, 1967). In VR rehabilitative treatments, implicit learning is often achieved using motor-cognitive dual-task training, and shown to enhance ML more efficiently in patients with neurological disorder (Fritz et al., 2015). Such tasks include walking while counting backwards, or walking while trying to avoid obstacles, and appear to be more effective than walking alone (Yang et al., 2008; Mirelman et al., 2016) while giving a more realistic approach in including the multiple processes (i.e., motor, cognitive, and social) necessary for daily functioning (Faria et al., 2016, 2018; Perez-Marcos, 2018). Studies implementing VR motor-cognitive tasks in post stroke, Parkinson’s, and multiple sclerosis patients report significant improvements in both the motor and cognitive (memory, attention and visual-spatial abilities) domains (Maggio et al., 2019), with effects in the motor domain reportedly retained at follow-up (Mendes et al., 2012; Mirelman et al., 2016; Cano Porras et al., 2018; Faria et al., 2018).

Task variation is introduced by varying the difficulty of a performed task. Once the simple tasks are accomplished, complex tasks are introduced. This gradual level of learning sanctions a sense of triumph over the task, promoting self-efficacy and ML while increasing patients’ motivation and enjoyment (Levac et al., 2019). For example, Jaffe et al. (2004) changed the difficulty level of their task by increasing the length and height of the obstacles in a virtual training course while poststroke hemiplegia patients walked on a treadmill. While, Yang et al. (2008) increased the speed of the treadmill 5% after each training session, and introduced different walking scenarios. Such VR programs provide a more affluent training environment that involves adapting to unpredictable scenarios, which more reflect real-life scenarios more.

Task-specific training is one of most important aspects for treatment rehabilitation as it postulates that ML is promoted when the acquired movement skills are as close as possible to those expected to perform the task in the real world (Levac and Sveistrup, 2014). VR is the ideal candidate for such practice as it can simulate daily living challenges in a safe environment that with time translate to the real world. For example, Rand et al. (2009) placed post stroke patients in a virtual shopping mall, and measured multitasking abilities over a period of 3 weeks. Patients improved their multitasking abilities from 20.5 to 51.2% following this VR intervention.

Finally, real-time feedback is extremely important in ML as it provides some information as to how a task is being performed allowing the possibility to adapt the training accordingly, and reinforce movement control and reduce movement compensation (Subramanian et al., 2013). In a recent study, Van Gelder et al. (2017) measured gait performance of children suffering from cerebral palsy as they walked on a VR instrumented treadmill. They performed three conditions: one condition provided no feedback while the other two conditions provided feedback on hip and knee angle. Significant improvements were observed in hip and knee extensions following real-time feedback. Whereas, Mirelman et al. (2009) used real time feedback to encourage their patients whenever they successfully navigated their target by changing the target color from yellow to green along with the word “GREAT.” Providing VR real-time feedback provides patients with the opportunity to become aware of their shortcomings, as well as, their progress, motivating them to continue towards the road to autonomy, improving activities in their daily living, and improving their overall quality of life.

Taken together, VR has the ability to implement all elements of ML and can improve performance of movement skills important for real world functioning in patients suffering from different neurological disorders.

## Discussion

To our knowledge, no study to date has utilized ML in VR specifically for motor rehabilitation in patients with schizophrenia. However, patients with schizophrenia have been mastering VR trainings in previous research (Valmaggia et al., 2016; Rus-Calafell et al., 2018). Specifically, VR studies using elements of ML outside the motor domain, show promising results in enhancing and maintaining interpersonal social skills, as well as, reducing auditory hallucinations and paranoia in schizophrenia patients (Rus-Calafell et al., 2018). VR settings designed to allow schizophrenia patients to interact with different virtual characters while encouraging progressive learning of social skills and providing both positive and negative reinforcement showed significant improvements in emotion perception, assertive and conversational behavior, as well as, negative symptoms, psychopathology, social avoidance, discomfort, and functioning (Park et al., 2011; Rus-Calafell et al., 2014; Adery et al., 2018). Most of these gains were also maintained at 4-month follow-up (Rus-Calafell et al., 2014). In addition, schizophrenia patients undergoing a 10 h VR job interview training significantly improved their virtual interview and role-playing performance scores across increasing levels of difficulty and had greater odds of receiving a job offer at 6-month follow-up (Smith et al., 2015). Furthermore, VR therapy designed to have schizophrenia patients confront and interact with a visual representation of their most distressed auditory hallucination produced significant improvements in auditory verbal hallucination severity, depressive symptoms, as well as, quality of life that remained at 3-month follow-up (Du Sert et al., 2018), while, error-feedback during social perception judgments reduced paranoid ideation in patients with schizophrenia (Moritz et al., 2014).

Overall, these studies show VR’s efficacy and feasibility in improving symptoms associated with schizophrenia. The use of ML elements in these paradigms shows patients’ ease in responding and adapting to the ever changing and increasingly challenging virtual environments reinforcing overall learning outcome. Patients have also recognized their enjoyment and increase motivation when using VR therapy where its use in combination with conventional therapy can have significant and everlasting benefits that can greatly influence patients’ quality of life (Adery et al., 2018; Du Sert et al., 2018; Rus-Calafell et al., 2018).

Since, schizophrenia patients respond well to VR-therapy, and are able to adapt to different scenarios, we can apply ML in VR to ameliorate movement abnormalities in these patients by restoring, retaining, and transferring the learned movement skills, similarly to that done with neurological patients. Why is it important to do so? Movement abnormalities are ubiquitous across all stages in schizophrenia patients. These movement deficits are linked to socio-cognitive impairments, such as gesture performance, an integral part of communication, which can have devastating consequences on patients clinical outcome and overall functioning (Walther et al., 2020d). Designing a VR paradigm that encompasses motor and socio-cognitive domains, where for example, communicative gestures are mastered and are then applied during social interactions, can improve patients’ overall communication. This can have substantial benefits in how they express themselves to their therapist or doctor, how they navigate daily tasks, while promoting autonomy and overall functioning.

## Future Outlook and Conclusion

Although movement abnormalities are prevalent in schizophrenia affecting overall communication and social functioning very little has been done to treat and alleviate these deficits. Psychopharmacology has proven to have very little effects on psychomotor abnormalities, while NIBS has some promise (Lefebvre et al., 2020). In this perspective paper, we highlight VR’s success in effectively combining all elements of ML in improving motor abnormalities in neurological disorders, and advocate its potential use in ameliorating, restoring, and improving movement abnormalities in schizophrenia patients. Using ML, we can combine motor and socio-cognitive domains to establish personalized simulated real-life scenarios tailored to each patient’s individual needs promoting autonomy that can greatly improve their quality of life, such as gesture performance. In addition, combining VR with NIBS can further benefit patients with schizophrenia as, neuromodulation of the affected cortical areas while being placed in a safe virtual environment, could allow for direct translation to the real world (Gainsford et al., 2020; Lefebvre et al., 2020).

## Data Availability Statement

The original contributions presented in the study are included in the article/supplementary material, further inquiries can be directed to the corresponding author.

## Author Contributions

AP drafted the manuscript. SW revised the manuscript. AP and SW edited the manuscript. Both the authors contributed to the article and approved the submitted version.

### Conflict of Interest

SW received honoraria from Janssen, Lundbeck, and Sunovion. AP declares no conflict of interest.
